# Rearing and Maintenance of *Galleria mellonella* and Its Application to Study Fungal Virulence

**DOI:** 10.3390/jof6030130

**Published:** 2020-08-07

**Authors:** Carolina Firacative, Aziza Khan, Shuyao Duan, Kennio Ferreira-Paim, Diana Leemon, Wieland Meyer

**Affiliations:** 1 Molecular Mycology Research Laboratory, Centre for Infectious Diseases and Microbiology, Faculty of Medicine and Health, Sydney Medical School, Westmead Clinical School, Marie Bashir Institute for Infectious Diseases and Biosecurity, The University of Sydney, Westmead Hospital (Research and Education Network), Westmead Institute for Medical Research, Westmead 2145, NSW, Australia; cfiracative@gmail.com (C.F.); azizakhan0112@gmail.com (A.K.); sdua3794@gmail.com (S.D.); kenniopaim@gmail.com (K.F.-P.); 2 Studies in Translational Microbiology and Emerging Diseases Research Group (MICROS), School of Medicine and Health Sciences, Universidad del Rosario, Bogota 111221, Colombia; 3 Infectious Disease Department, Triangulo Mineiro Federal University, Uberaba 38025-440, Brazil; 4 Agri Science Queensland, Department of Agriculture and Fisheries and Forestry, Brisbane 4102, QLD, Australia; Diana.Leemon@daf.qld.gov.au

**Keywords:** *Galleria mellonella*, *Cryptococcus gattii*, larvae, fungal infection, animal model

## Abstract

*Galleria mellonella* larvae have been widely used as alternative non-mammalian models for the study of fungal virulence and pathogenesis. The larvae can be acquired in small volumes from worm farms, pet stores, or other independent suppliers commonly found in the United States and parts of Europe. However, in countries with no or limited commercial availability, the process of shipping these larvae can cause them stress, resulting in decreased or altered immunity. Furthermore, the conditions used to rear these larvae including diet, humidity, temperature, and maintenance procedures vary among the suppliers. Variation in these factors can affect the response of *G. mellonella* larvae to infection, thereby decreasing the reproducibility of fungal virulence experiments. There is a critical need for standardized procedures and incubation conditions for rearing *G. mellonella* to produce quality, unstressed larvae with the least genetic variability. In order to standardize these procedures, cost-effective protocols for the propagation and maintenance of *G. mellonella* larvae using an artificial diet, which has been successfully used in our own laboratory, requiring minimal equipment and expertise, are herein described. Examples for the application of this model in fungal pathogenicity and gene knockout studies as feasible alternatives for traditionally used animal models are also provided.

## 1. Introduction

The greater wax moth *Galleria mellonella* (Lepidoptera: Pyralidae) is a ubiquitous pest of honeybee colonies globally causing damage to wax combs in stressed beehives and stored beekeeping equipment, where the larvae feed and transform into moths [1]. *G. mellonella* can undergo a complete life cycle in 8 to 12 weeks under favorable conditions of temperature and humidity [1]. Eggs hatch to larvae, which undergo 7 moults (instars) before pupation and metamorphosis into adults. The cream-colored 6th instar larvae, which are about 3 cm in length and weigh approximately 300 mg, have become increasingly popular as an alternative non-mammalian model to study fungal disease and virulence [2,3,4,5,6,7]. As a model host, *G. mellonella* larvae have several advantages including low maintenance costs, ability to obtain large quantities, ease of fungal inoculation, and use without major ethical constraints. These larvae can be kept at a range of temperatures, from 20 °C to 42 °C, allowing experimental procedures to mimic mammalian physiological conditions. Moreover, previous studies have demonstrated that results obtained by the fungal inoculation of larvae correlate with those obtained with murine model experiments [7,8].

Currently, no standardized procedures and conditions for maintenance of the larvae are available. Although they can be obtained commercially in some countries, the propagation protocols, dietary conditions, humidity, and temperature for their maintenance and anti-bacterial requirements differ among companies [9,10]. In addition, different research groups have also reported various dietary conditions for the larvae that are being tested, namely, starvation for one week before inoculating versus no starvation, and providing food after inoculation versus no food provided after inoculation [9]. Furthermore, the insect is not readily available for purchase in most countries and ordering from overseas suppliers can cause the larvae stress due to transportation conditions, resulting in altered larval immunity. These factors would decrease the reproducibility of virulence experiments using *G. mellonella* among laboratories [11,12]. It has also already been demonstrated that batches of larvae from different suppliers or even from the same supplier can vary in their genetics and overall health, thus affecting larval survival rates [10]. In addition, it may not be possible to obtain larvae from overseas suppliers due to import restrictions. Therefore, propagating and maintaining *G. mellonella* larvae within the laboratory will be useful for ongoing fungal virulence studies, while also allowing the larvae to acclimatize to optimal conditions for one or more life cycles before experimental use.

The successful use of *G. mellonella* as a reproducible model for fungal virulence experiments relies heavily on the standardization of maintenance and propagation methods to produce the least genotypic and phenotypic variability, which is best achieved by maintaining larval cultures within a controlled laboratory environment. This will overcome any effects due to the variable methodology used by suppliers and also provide a regular, consistent diet to produce larvae of reliable quality for fungal virulence experiments.

Here, simple and cost-effective methods for rearing *G. mellonella* through its life cycle in a laboratory using only basic equipment, even with no prior insect handling experience, is established. These methods have proven to be successful in our own laboratory where we have continually cultured over 10 years approximately 45 generations of *G. mellonella*. The inoculation protocol associated with the larvae and its advantage as a model for studying fungal infection and virulence, specifically in the example to study cryptococcosis, a life-threatening fungal infection of the lungs and the central nervous system (CNS), is also described.

## 2. Protocols

### 2.1. Larvae

The larvae were originally obtained from the wax moth culture maintained by the Queensland Department of Agriculture and Fisheries at the Ecosciences Precinct in Dutton Park, Brisbane, Australia.

### 2.2. Housing Containers and Storage Conditions

**Figure 1:**
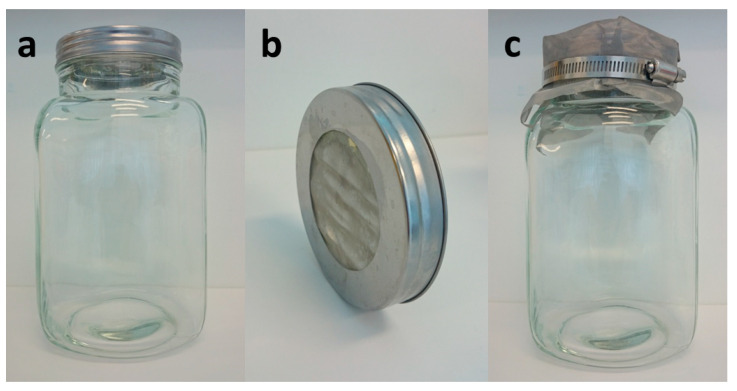
Storage containers for rearing and maintaining the larvae. Glass housing jar approximately 22 cm height with wide neck for housing larval colonies (**a**). Modified jar lid (**b**). Ultra-thin stainless-steel wire mesh is fixed by a hose clamp around the lid as a secondary layer (**c**).

Tall wide neck glass jars (approximately 22 cm high and 10 cm in diameter) were used for rearing the larvae (Figure 1a). Prior to culturing, jars were sterilized by autoclaving to prevent mold and bacterial growth. Jar lids were modified by cutting a large hole through the center and replacing it with ultra-thin stainless-steel wire mesh (sieve size of 0.1 mm^2^) to allow ventilation (Figure 1b). Any wooden, plastic, or cotton material was avoided to store larvae as they can chew through, allowing escape. A piece of ultra-thin stainless-steel wire mesh secured by a perforated hose clamp was used to cover the whole lid as a secondary protective layer (Figure 1c). Overall, the containers were well ventilated and of a clear material allowing observation while being able to contain young larvae (around 600 larvae per jar). Colonies were sporadically stressed by mold or bacterial growth. In this case, the affected larvae (presenting increase in melanin production and/or slow movements) and the contents of the glass jars were disposed of appropriately, with jars washed and autoclaved before reuse.

The time taken for *G. mellonella* to complete its life cycle is affected by temperature. It can take 8–10 weeks at temperatures between 28 and 34 °C (Table 1), but up to 13 weeks at room temperature (24 °C). Larvae and pupae were reared and incubated in the insectarium of the Westmead Hospital Animal Care Facility, Sydney, Australia, under controlled conditions of 26 °C and 60% relative humidity. The housing equipment was kept in a relatively dark area where they would not be disturbed aside from feeding and larval collection periods.

### 2.3. Culture Medium for G. mellonella

The following protocol made culture medium (artificial diet) for approximately 1800 larvae (or three housing jars). In a sterilized beaker, 58.3 g (22%) of glycerol, 58.3 g (22%) of organic honey, and 10 mL (4%) of water were combined. Then, the mixture was heated in a microwave at 1000 W for 1 min and allowed to cool to room temperature. Then, 250 g (48%) of cereal was mixed with the liquid until the mixture crusted; then, 8 g (4%) of instant dry baker’s yeast were added and mixed thoroughly. Farex^®^ Original multigrain cereal—fine grains, 6+ months, H.J. Heinz Company, Southbank, Victoria, Australia is recommended. If not available, an equivalent product containing a mixture of ground rice, maize flour, soy flour, vitamins [vitamin C, Niacin (B3), Thiamin (B1)], mineral (iron), and traces of wheat and milk could be used. The culture medium was prepared fresh and not stored, as this dried the mixture.

### 2.4. Rearing of G. mellonella in the Laboratory

After obtaining the larvae, they were placed into glass housing jars with fresh culture medium filled up to 5–8 cm high, and the 6th instar mature larvae that were producing silk were allowed to form a protective cocoon and transform into pupae. At this stage, feeding of the larvae stopped. Several jars of colonies were always kept, to prevent loss in production due to mold or bacterial contamination, which occurred seldomly. When the adult moths emerged, up to 10 moths were transferred to a separate housing jar with fresh medium, using a male to female ratio of 1:1. To prevent the moths from flying away, they were transferred to new jars by making them fly into a plastic bag, which was attached to the jar before the lid was open. Male moths have a wingspan of 10 to 15 mm and have a lighter color with faint markings. Adult female moths are larger with a wingspan of up to 20 mm and are of a darker brown coloration (Table 1). Note that the moth stage does not require feeding and females will die after laying eggs.

Adult female moths began laying eggs for 1 to 2 weeks at 26 °C. Eggs could be found at the edge of the lid or rim of the jar and were scraped gently with a scalpel blade and dropped into the culture medium. When newly laid eggs did not lift off the surface easily, one more day was left before moving them. Eggs hatched in 1 to 2 weeks, and tiny larvae were gravitating to the bottom of the glass jar where they could be seen on their thin trail marks. All moths were discarded once hatched larvae were evident in the same housing jar. Live moths were disposed by placing them into plastic bags, which were stored for one day in a fridge and then took for incineration. Female moths can lay 400 to 1000 eggs at a time.

Larvae were reared for 5 to 6 weeks, during which fresh culture medium was added twice weekly. Separating larvae into new jars ensured an appropriate ratio of food per larvae and avoided overcrowding, which can cause stress and the discoloration of larval cuticles. Old and hardened medium was discarded regularly. Lids were frequently cleaned of webbing and pupating larvae to improve cross-ventilation. *G. mellonella* pupated for 2 to 3 weeks, and a new cycle began when adult moths emerged.

Sixth instar larvae, which were lightly colored with no graying or dark marks visible and weighed around 300 mg and were 3 cm long, were selected for inoculation (see below).

### 2.5. G. mellonella Inoculation

To assess the performance of *G. mellonella* larvae as a model system to study fungal virulence, three *Cryptococcus gattii* strains previously studied in a murine model of infection were selected for inoculation into *G. mellonella* larvae reared and maintained in our insectarium. The tested strains were CDCR265 (VGIIa) and CDCR272 (VGIIb) from the Vancouver Island outbreak, which have been characterized as highly and low virulent, respectively [13], and the strain DMST20767, which was previously found to be of comparable virulence to CDCR265 in a murine model [14].

To prove the value of *G. mellonella* larvae as a screening tool for the impact of gene knockouts on virulence profiles, two samples of knockout mutants were also tested: the is-cDNA 26, which corresponds to the putative collagen binding domain of a collagenase gene present in the Pacific North West VGIIa genotype, and the is-cDNA 2, which corresponds to the endoribonuclease gene present in the VGIIa/c genotypes [15]. These genes were disrupted in the high-virulence reference strain CDCR265 using overlap-PCR with the selective marker nourseothricin acetyltransferase (NAT) [16] and transformed using a biolistic shotgun to generate knockout mutants by homologous recombination. Both knockouts Δ*coll* and Δ*endo* were compared with the wild-type CDCR265 strain in the *G. mellonella* larvae.

**Figure 2:**
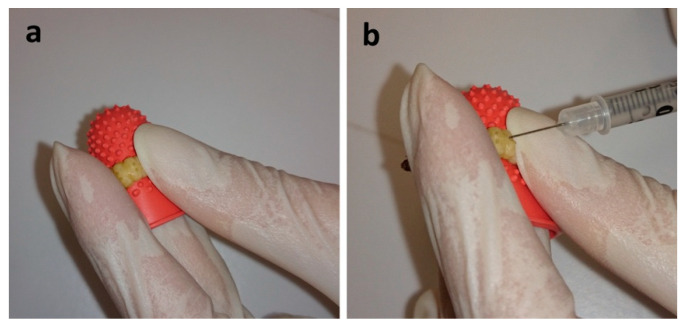
Inoculation of the larvae. Exposure of the pro-legs over the middle finger (**a**). Inoculation of the larvae into the last left pro-leg (**b**).

Groups of 10 similar-sized 6th instar larvae were selected and placed in a Petri dish. Fungal strains were grown on Sabouraud’s Dextrose agar at 27 °C for 48 h prior to inoculation. The yeast inoculums were prepared in phosphate-buffered saline (PBS), and the concentration was adjusted to 1.0 × 10^8^ cells/mL using a Neubauer chamber. Injections were performed with 10 µL of the inoculum using a 50 U insulin syringe with a 29-gauge needle. For inoculation, each larva is held over the middle finger and protected with a rubber thimblette, exposing the larval pro-legs (Figure 2a). The forefinger and thumb are used to stabilize the insect and create a bend for the syringe needle to enter the last left larval pro-leg (Figure 2b). Ten uninoculated and 10 PBS-inoculated larvae were included as controls to ensure that environmental conditions and physical injuries by inoculation, respectively, do not affect the survival rates. Post inoculation, larvae were placed into a clean Petri dish without food and incubated at 37 °C. Mortality was monitored at 24-h intervals over a period of 10 days by gently probing the larva for movement. Dark coloration of the cuticle was also monitored for severity of infection. Survival curves were graphed and analyzed by Log-Rank (Mantel–Cox) test using the Graph Pad Prism 6.04 software (La Jolla, CA, USA), and median survival times were obtained for each strain.

### 2.6. Histopathology

At the end point of the experiment, dead infected larvae were collected and immediately placed in 10% buffered formalin fixative for a minimum of four weeks. The long fixation period produced better results, since the larval exoskeleton has low permeability to most fixative reagents [17]. Then, the larvae were transferred into increasing concentrations of ethanol (70%, 80%, 90%, and 100%) for one hour each, embedded in paraffin, cross-sectioned, and stained by histochemical techniques with hematoxylin and eosin (HE) and special stain Mayer’s Mucicarmine (MM) to stain the cryptococcal cell capsules red. Photographs were taken of the prepared slides using an Olympus BX43 microscope.

## 3. Results

**Figure 3:**
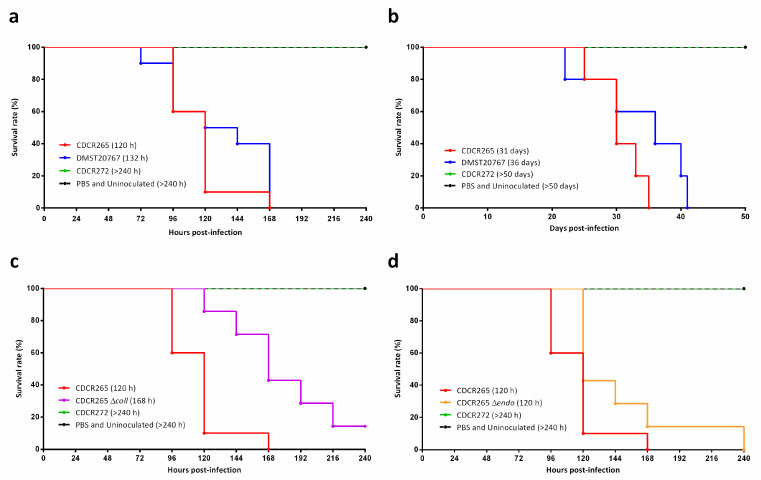
Log-Rank survival curves and median survival times (MST) of 10 *Galleria mellonella* larvae (**a**) and six female BALB/c mice based on data previously obtained by our group [13,14] (**b**) inoculated with different *Cryptococcus gattii* strains. *G. mellonella* inoculated with the knockout mutant strains Δ*coll* (**c**) and Δ*endo* (**d**) were compared with the high-virulence strain CDCR265 and the low-virulence strain CDCR272. The MST for each strain is given in brackets. Larvae (**a**,**c**,**d**) and mice (**b**) inoculated with phosphate-buffered saline (PBS) and uninoculated controls are shown (dotted line).

Results from the murine experiments were obtained from our previous study [14]. To show the capacity of the *G. mellonella* larvae model to study fungal virulence, the larvae were inoculated with the high-virulence *C. gattii* strain CDCR265, which began dying 96 h after inoculation, and with the strain DMST20767, with which the larvae began dying 72 h post inoculation (Figure 3a). However, the median survival times calculated indicated that statistically, there was no significant difference between the survival curves for the two strains (*p* = 0.2841), which was similar to the results obtained in mice inoculated with these two strains (*p* = 0.1375) (Figure 3b) [14]. Strain CDCR272 did not cause mortality in either *G. mellonella* larvae or mouse models (Figure 3a,b).

To show the applicability of the *G. mellonella* larvae model to characterize mutant strains, the *C. gattii* knockout strains ∆*coll* and ∆*endo* were screened for enhanced, reduced, or similar pathogenicity in *G. mellonella* larvae compared to the wild-type strain CDCR265. The survival curve for ∆*coll* showed a significantly lower virulence (*p* = 0.0030) (Figure 3c), whilst ∆*endo* was found to be of equal pathogenicity in comparison to the wild-type strain CDCR265 (*p* = 0.0645) (Figure 3d).

**Figure 4:**
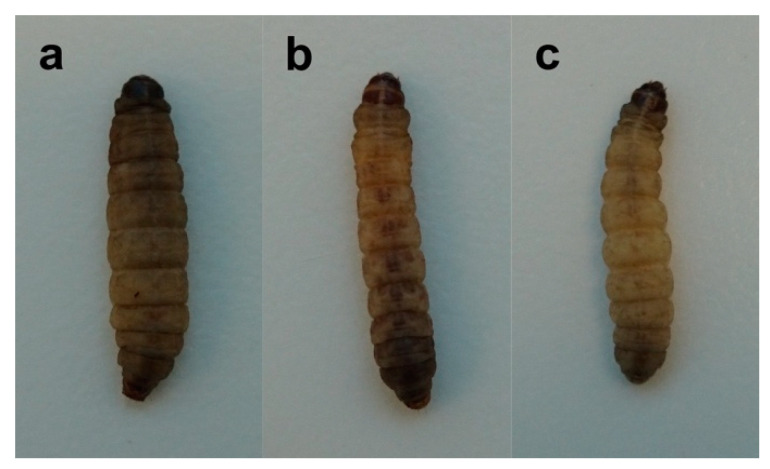
Larvae showing high melanization at day 6 post infection with *Cryptococcus gattii* strains CDCR265 (**a**) and DMST20767 (**b**), and little melanization of infected larva with CDCR272 (**c**).

Melanization is a primary immune response to infection in *G. mellonella* larvae and gives an indication of the severity of infection [1]. Strains CDCR265 and DMST20767 showed similar degrees of melanization five days post infection (Figure 4a,b). Larvae infected with the low-virulence *C. gattii* strain CDCR272 presented higher median survival times with little to no melanization in comparison to the high-virulence strains (CDCR265 and DMST20767), as expected (Figure 4c).

**Figure 5:**
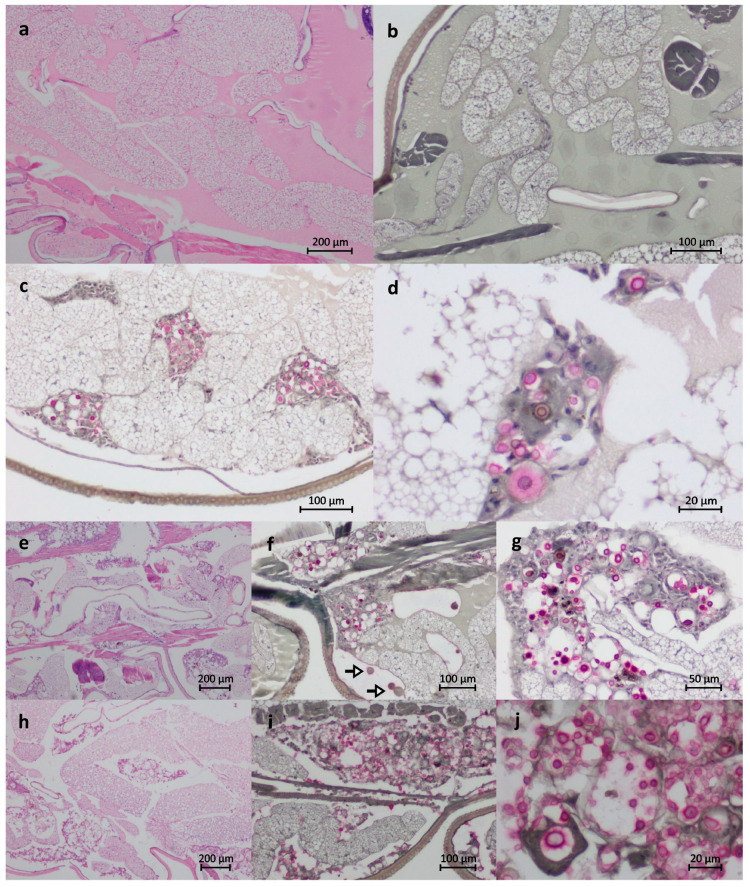
Hematoxylin and eosin (HE) and Mayer’s Mucicarmine (MM) stained sections of *Galleria mellonella*. Uninfected control larvae stained with HE (**a**) and MM (**b**) are shown. MM stained larvae infected with the low-virulence *Cryptococcus gattii* strain, CDCR272, show cryptococcal cells contained within small nodular lesions surrounded by hemocytes (**c**,**d**). Little muscle damage apart from nodular regions is seen. HE (**e**) and MM (**f**,**g**) stained larvae infected with the high-virulence *C. gattii* strain, CDCR265, show skeletal muscle damage and large nodular lesions. Giant cryptococcal cells, which are too large to be engulfed by hemocytes, are indicated by arrows. HE (**h**) and MM (**i**,**j**) stained larvae infected with the strain DMST20767 show extensive skeletal muscle damage. Cryptococcal cells are found in nodular regions and diffused throughout the surrounding tissue and hemocoel.

Histopathological findings of uninfected and infected larvae indicated that skeletal muscle damage and high cryptococcal cell numbers and spread within the organism are correlated with virulence (Figure 5a–j). The larvae infected with the high-virulence strains CDCR265 and DMST20767 showed skeletal muscle damage and large nodular lesions (Figure 5h–j), while the larvae infected with the low virulent strains CDCR272 shows little muscle damage and small nodular lesions (Figure 5c–g).

## 4. Discussion

The use of non-mammalian models in virulence studies overcomes important limitations that come with murine models, including ethical considerations associated with mammalian model systems, housing expenses, and time spent on maintenance. To use the model in an ethical way, the number of larvae used per experiment should still be carefully considered. The *G. mellonella* insect model has proven useful in numerous fungal pathogenesis studies that include yeasts, such as *Candida* spp. [18,19] and *Trichsoporon* spp. [20]; molds, such as *Aspergillus* spp. [6,21], *Fusarium oxysporum* [22], *Scedosporium aurantiacum* [23], *Madurella mycetomatis* [24], and Mucorales species [25], as well as the dimorphic fungi *Histoplasma capsulatum* and *Paracoccidiodes lutzii* [26]. Moreover, the *G. mellonella* model has the additional advantage of being inexpensive to propagate within the laboratory in comparison to mammalian models and easy to manipulate for experimental procedures. In addition, apart from studying the pathophysiology of different fungal species, more recently, this invertebrate model has been successfully used for testing the in vivo efficacy of conventional and novel antifungal drugs [27]. In the case of *Cryptococcus neoformans* and *C. gattii*, several approaches using mainly vertebrate but also invertebrate models have been used to study fungal pathogenicity [10,28,29,30,31,32,33], recognize genes involved in pathogenicity, identify strain virulence, virulence factors (including capsule, melanin production and biofilm formation), and undertake antifungal susceptibility testing of existing and new compounds [34,35,36,37,38,39].

Although wax moth larvae are a very common pest found in apiaries, especially on old wax combs in storage, and it may be possible to source the initial larvae to set up a moth breading colony directly from a beekeeping operation, for laboratories routinely ordering *G. mellonella* larvae for ongoing experiments, there are likely to be a range of issues. These may include the cost, availability, and permissions requiring importation and transportation in unfavorable conditions, which can affect insect survival rates. To overcome these issues, cost-effective and simple methods for the propagation and maintenance of *G. mellonella* using inexpensive equipment (Table S1), with minimal expertise, were developed. The larval cultures are self-contained, with development from egg to adult moth in the same environment allowing a continuous supply of larvae from multiple jars of *G. mellonella* at various life stages (Table 1). In addition, the diet described here took into account the results reported in a previous study, which compared three different dietary conditions [40], but simplified even more the diet composition, still containing all nutrients required for the healthy and fast growth of the larvae, including carbohydrates, protein, and lipids, as previously reported. Using the herein described rearing method, the *G. mellonella* colony has been continuously maintained for approximately 45 life cycles since the colony was started in 2011.

This report also provides an artificial diet protocol for researchers who have ordered larvae for experimental use within 1 to 2 weeks. Most studies suggest utilizing such larvae within 7 days of receiving them. However, if this is not possible, our protocol can be used to provide sustenance for the larvae. It has been shown that larvae deprived of food for 7 days have a reduced expression of a range of antimicrobial peptides and immune proteins causing an increased susceptibility to infection [11]. Other factors, such as temperature, agitation, and transportation conditions have also been shown to increase stress and infectivity [10,12,18,40]. This may result in a variation in results between laboratories working with the same fungal strains, thus highlighting the importance of having standardized procedures for both the maintenance of larvae to allow acclimatization and subsequent experimental protocols, as reported previously [9].

*G. mellonella* larvae are relatively large in size, which facilitates easy inoculation and the collection of hemolymph and tissue samples for further analysis. Training to perform systemic inoculation of the larvae takes a few hours. Therefore, it is suggested to first practice the inoculation procedure with PBS. Other methods for the delivery of fungi have been previously described [5]. However, injecting offers the benefit of direct delivery of known fungal cell concentration into the larval hemocoel [4,41].

There are a number of advantages for using the *G. mellonella* model in fungal virulence studies. They are easily maintained under temperatures from 25 to 37 °C, and they are also suitable to mimic the physiological conditions in humans and other mammals, enabling temperature-related virulence studies [7,42]. By contrast, other non-mammalian models currently used for fungal studies do not possess this advantage and can only survive at temperatures lower than 27 °C [41]. However, not all fungal virulence factors necessary for the pathogenesis of human fungal infections are activated below human physiological temperatures. For example, *C. neoformans* demonstrates a higher degree of virulence at 37 °C than at 30 °C in larvae. Many of the same virulence traits involved in mammalian pathogenesis (such as polysaccharide capsule and various *C. neoformans* genes; *GPA1*, *PKA1*, and *RAS1*) were also associated with larval mortality [7]. Furthermore, in many aspects, the immune response against pathogens in insects is similar to the innate immunity in mammals [38,43]. Hemocytes in *G. mellonella* larvae use a similar mechanism to human neutrophils to recognize fungal cells using factors, such as pathogen recognition receptors, phagocytosis, and the production of superoxide, in the oxidative burst pathway [44]. The structural and functional similarities of the larval innate immune system to mammalian innate immunity makes *G. mellonella* an exceptional screening model for fungal disease, phagocytic cell studies, and screening antifungal agents with results correlating to those obtained in mammalian models [20,22]. To show two examples for potential applications of the *G. mellonella* model, we have applied it to fungal virulence and gene knockout impact studies.

Our own virulence experiments showed comparable results, using survival curves (Figure 3), degree of melanization (Figure 4), and histopathology (Figure 5) of larvae infected with *C. gattii* strains that have been previously characterized using the BALB/c mice model [13,14]. The strains CDCR265 and DMST20767 were highly virulent, and strain CDCR272 was low virulent in both the *G. mellonella* larvae (data obtained herein) and the mouse model (data previously obtained by our group [13/14]). However, the main differences between both models is the duration of the experiments, with the *Galleria* model cutting down the time to less than two weeks, while the mouse model takes almost two months (Figure 3). Similarly, larval tissue damage was more evident in larvae inoculated with high-virulence strains, as seen previously in murine models, in which rodents inoculated with strains of higher virulence presented a higher fungal burden [31,32,33]. Skeletal damage at the tissue level and nodular lesions consist typically of an accumulation of yeast masses that can be observed causing tissue disorganization, which resembles the formation of granulomas or lesions in the brain parenchyma in pulmonary or meningeal cryptococcosis, respectively [45].

The pathogenicity of fungal knockout mutant strains has also been successfully assessed in the *Galleria* larvae model, and these strains have been found to have similar virulence when tested in murine models [8,21]. Here, the low virulence of the knockout mutant strain Δ*coll* was probably related to a weak attachment in the larva soft tissues, as collagen is an essential protein widely distributed among living organisms, including *G. mellonella*. Pre-treatment of *G. mellonella* with peptides that inhibit the cell wall adhesion of *Paracoccidiodes brasiliensis* and *P. lutzii* increased the survival of larvae from 64% and 60% respectively, in addition to the hemocytes count [46]. The lack of difference in the knockout mutant strain Δ*endo* could be related with redundancy in the pathways controlled by this large family of proteins. Virulence experiments with fungal mutants in larvae can also assist in the selection of mutant strains for further analysis in mammalian models, thereby reducing the number and unnecessary killing of experimental animals, as well as obtaining preliminary data for funding applications. However, the *G. mellonella* larvae model does not give insights into the adaptive immune response with regard to antibody generation and cannot be used to model organ specific pathologies [4]. Although the first draft genome sequence of *G. mellonella* was reported in 2018 [47], studies on transcriptional genetics and complex innate immune responses and homology studies between *G. mellonella* and human, mouse, and other model hosts are still needed. Thus, this model is still used best for screening virulent fungal strains, knockout mutant strains, or antifungal agents for subsequent mammalian model experiments [3,8,21,28]. Finally, a recent study described some variation between the results obtained using the *G. mellonella* model when compared with the traditionally used mouse model and concluded that it does not predict murine survival for all studied strains, indicating that some caution should be used when interpreting the results [48].

As the popularity of *G. mellonella* as a virulence model for both fungi and bacteria has increased, standardized propagation methods and experimental procedures are needed to make *G. mellonella* larvae a successful virulence model. In this study, this issue was addressed by describing simple methods for successfully rearing these *G. mellonella* larvae through all life cycle stages using an artificial diet and standardized conditions. Our protocols also provide quality *G. mellonella* larvae with the minimal genetic and phenotypic variability needed for reproducible virulence studies.

## Figures and Tables

**Table 1:** Summary of the life cycle of *Galleria mellonella* and maintenance requirements at each stage.

Stage	Life Span	Comments	Maintenance Requirements
Egg *	2 weeks	After mating, the adult female moths usually lay their eggs and die. A single female moth can lay as many as 1000 eggs in the medium and on container walls. Eggs will then hatch at ambient temperature.	Once eggs are hatched, discard all dead adult moths as well as pupating larvae that have not emerged as moths.
Larva 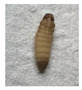	5 to 6 weeks	After eggs hatch, the young larvae feed on culture medium and begin to produce their webbing/silk. Larvae use this silk for cocooning as a protective mechanism and for transforming into pupae. Healthy larvae are cream colored with no dark discolorations. Note that overcrowding in the jar can cause stress, resulting in infection with mold or other microorganisms causing gray markings and pigmentation on the larvae.	It is important to feed young larvae twice weekly to ensure a maximum number of healthy non-pigmented larvae that can be used for experiments. It is advised to divide larval colonies of overcrowded jars into separate new jars. Lids should be cleaned of webbing frequently to improve ventilation. Once they develop into mature larvae (2–3 cm in length) place healthy, cream-colored larvae into a separate jar with fresh medium in preparation for experimental use.
Pupa 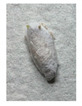	2 to 3 weeks	Mature larvae start to spin cocoons then remain in the pupal stage until ready to emerge as adult moths. At this stage, they will no longer consume food and live off the fat supplies in their bodies.	When the cocoons are not easily cut open, the larvae are pupating and should not be used in experiments as this causes bias in survival rates. No feeding is required after all larvae in the jar have cocooned.
Adult 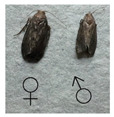	2 weeks	The adult moths do not feed. Females will usually begin laying eggs within a few days after they emerge.	Transfer moths at a male to female ratio of 1:1 into a separate jar with fresh medium. Check once weekly for eggs on sides of the jar and bottom of lid.

* Eggs are not pictured as they are too small to see.

## Supplementary Materials

The following are available online at https://www.mdpi.com/2309-608X/6/3/130/s1, Table S1: Equipment and average cost involved in propagating *Galleria mellonella* in the laboratory.
