# Supplementary material for: Rearing and Maintenance of Galleria mellonella and Its Application to Study Fungal Virulence
Source: J Fungi (Basel). 2020 Aug 7;6(3):130. doi: 10.3390/jof6030130 (PMC7558789; doi:10.3390/jof6030130)
Supplement: Supplementary file 1 [file jof-06-00130-s001.pdf]

Journal of Fungi

MDPI

Supplementary Materials: Rearing and Maintenance of *Galleria mellonella* and its Application to Study Fungal Virulence

Table S1. Equipment and average cost in US dollars involved in propagating *Galleria mellonella* in the laboratory.

Expenditure	Item	Cost	Total One-Off Expense	
**One-off**	Glass housing jar (minimum 3 required)	$5.70	$36.80	
	Ultra-thin stainless-steel wire mesh, sieve size 0.1 mm^2 (per m^2)	$17.50		
	Perforated hose clamp seal (78–102 mm)	$3.60		
**Recurring**	**Item**	**Cost**	**Total Cost for One Feeding**	**Total Cost per Month**
	Glycerol (58.3 g)	$2.20	$5.30	$42.40
	Organic honey (58.3 g)	$0.80		
	Farex® Original multigrain cereal—fine grain, 6+ months (250 g)	$2.10		
	Baker's Yeast (8 g)	$0.20		
